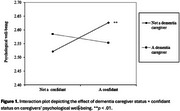# Dementia and Nondementia Caregivers Serving as Confidants: Implications for Their Psychological Well‐Being

**DOI:** 10.1002/alz70858_097262

**Published:** 2025-12-24

**Authors:** Meng Huo, Fei Wang

**Affiliations:** ^1^ UC Davis, Davis, CA, USA; ^2^ The University of Tennessee Knoxville, Knoxville, TN, USA

## Abstract

**Background:**

Caregivers are typically a mainstay of help with activities of daily living when older adults experience health concerns, but some caregivers also serve as confidants for discussing private matters. A burgeoning literature has revealed the health benefits of having at least one caregiver who also is a confidant. It remains unclear, however, who those caregivers are and how serving additional confidant roles is associated with their psychological well‐being. The current study addressed these gaps and, considering the unique experience of dementia caregiving, compared between dementia and nondementia caregivers.

**Method:**

We used the 2017 survey data from the *National Health and Aging Trends Study* and the supplemental *National Study of Caregiving*. Participants included 2,652 caregivers (*M_age_* = 61.61 years old; 916 dementia caregivers, 1,736 non‐dementia caregivers) of 1,697 older adults aged 65+ (i.e., care recipients). Care recipients nominated up to five confidants with whom they “talked most often about important things.” Caregivers reported their own demographic characteristics (e.g., age, gender, education, health, positive and negative relationship quality, relationship type) and psychological well‐being (e.g., feeling cheerful, calm, full of life). We conducted two‐level models as caregivers were nested within care recipients.

**Result:**

Caregivers who were older, female, healthier, spouses or children, and reported more positive as well as more negative relationships with care recipients were more likely to be identified as confidants. Post‐hoc pairwise comparisons further revealed that spousal caregivers were mostly likely also confidants, followed by adult child caregivers, and then other types of caregivers. These associations remained the same for both dementia and nondementia caregivers. Caregivers who were confidants reported better psychological well‐being than those who were not, but this association varied by dementia caregiving status and was only evident among non‐dementia caregivers (see Figure 1).

**Conclusion:**

This study extends prior work by revealing how serving multiple roles affects caregivers’ psychological well‐being and corroborating differences between dementia and nondementia caregiving. More research is needed to better understand the unique stressors dementia caregivers may be exposed to when serving as confidants for their care recipients, and interventions should be customized to allocate targeted resources to caregivers faced with different demands.